# Aims of the Adherence Promotion With Person-Centered Technology (APPT) Project

**DOI:** 10.1093/geroni/igab046.2116

**Published:** 2021-12-17

**Authors:** Neil Charness, Walter Boot, Dawn Carr, Shayok Chakraborty, Zhe He, Mia Lustria, Antonio Terracciano

**Affiliations:** 1 Florida State University, Tallahassee, Florida, United States; 2 School Of Information, Florida State University, Florida, United States; 3 FLORIDA STATE UNIVERSITY, Florida State University, Florida, United States

## Abstract

The APPT project supports the early detection and treatment of age-related cognitive decline and dementia by 1) enhancing adherence to cognitive intervention and assessment protocols, 2) improving understanding of barriers to long-term adherence, and 3) developing algorithms for predicting and preventing adherence failures. Two randomized controlled trials will test an adaptive technology support system predicted to boost adherence to cognitive protocols over a period of six months within samples of older adults with and without cognitive impairment. These studies will provide insight into the benefits of adherence support, and individual difference factors that should shape the adherence protocol, informing the process of identifying individuals who would benefit from additional support and predicting and preventing extended adherence failures before they happen. These studies should improve early detection and treatment of cognitive decline, extend functional independence, and improve lives of those with cognitive impairment as well as the lives of their families.

